# A short version of the reflective functioning questionnaire: Validation in a greek sample

**DOI:** 10.1371/journal.pone.0298023

**Published:** 2024-02-06

**Authors:** Evangelia Karagiannopoulou, Fotios S. Milienos, Alex Desatnik, Christos Rentzios, Vasileios Athanasopoulos, Peter Fonagy

**Affiliations:** 1 Department of Psychology, University of Ioannina, Ioannina, Greece; 2 Research Department of Clinical, Educational and Health Psychology, University College London, London, United Kingdom; 3 Department of Sociology, Panteion University of Social and Political Sciences, Athens, Greece; 4 Anna Freud National Centre for Children and Families, London, United Kingdom; 5 Open Door Young People Service, London, United Kingdom; 6 Department of Psychology, Neapolis University Pafos, Paphos, Cyprus; 7 Department of Education, University of Nicosia, Nicosia, Cyprus; Universita Cattolica del Sacro Cuore Sede di Roma, ITALY

## Abstract

This study aims to validate the Greek version of the 54-item Reflective Functioning Questionnaire (RFQ), a measure designed to assess an individual’s capacity for understanding themselves and others based on internal mental states. This capacity, also known as Reflective Functioning (RF) or mentalizing, is believed to play a significant role in both typical and atypical development. The validation process examined the factor structure of the RFQ and its relationship with a variety of psychosocial and clinical constructs that have theoretical and empirical links to RF. Additionally, this research investigated the factor structure’s invariance across gender and age groups to determine the robustness of the instrument. A unique contribution of this work lies in examining the application of the RFQ to attachment classifications through the use of cluster analysis. The sample consisted of 875 Greek adults from the general community with a mean age of 28.5 and a median age of 22. Participants completed the Greek RFQ along with a series of self-report questionnaires assessing psychosocial constructs, including attachment, epistemic trust, emotion regulation, and psychological mindedness, as well as clinical variables such as anxiety, depression, and borderline personality traits. Our findings suggest that a shorter, 31-item version of the questionnaire provides a robust three-factor structure across a non-clinical Greek adult population. The three identified subscales are (a) excessive certainty, (b) interest/curiosity, and (c) uncertainty/confusion, all demonstrating satisfactory reliability and construct validity. The uncertainty subscale was found to be associated with insecure attachment styles, epistemic mistrust and credulity, emotional suppression, and low psychological mindedness. In contrast, the certainty and curiosity subscales were linked to secure attachment, epistemic trust, emotion reappraisal, and psychological mindedness. Uncertainty was further shown to differ significantly across probable clinical and non-clinical groups, as distinguished by cut-off scores for anxiety, depression, and borderline personality disorder (BPD). However, the certainty and interest/curiosity subscales only varied between the two BPD groups. Our results provide the first evidence supporting the use of a 31-item version of the RFQ with three validated subscales to reliably assess reflective functioning in the Greek population, demonstrating stronger psychometric properties compared to other RFQ versions reported in previous studies. Findings suggest that impaired mentalizing capacity, as measured by the RFQ, is linked to insecure attachment, epistemic mistrust and credulity, poor emotion regulation, and low psychological mindedness, and potentially plays a role in adult mental health symptoms.

## Introduction

Mentalizing, operationalized as reflective functioning (RF), is the human capacity to interpret both the self and others in light of internal mental states such as feelings, desires, wishes, attitudes, and goals [1, 2]. This capacity is suggested to originate in the context of secure attachment relationships, fostered by a child’s experience of being mentally held and mirrored by a secure attachment figure [3].

On the other hand, insecure attachment relationships, likely interplaying with genetic and environmental vulnerabilities, have been linked with impaired mentalizing [4, 5]. Impairments in mentalizing have been associated with a broad spectrum of mental disorder symptoms, including but not limited to borderline personality disorder (BPD), depression, and anxiety [6–14].

Within this context, RF appears to mediate the relationship between insecure adult attachment and mental health or personality variables [15–18]. The mentalizing process enables individuals to perceive and label emotions related to difficult life experiences, as well as to reflect on these experiences, which may ultimately reduce their negative impact [19]. As a result, mentalizing is connected with critical psychological competencies such as emotion regulation [20, 21], distress tolerance [22], and the related construct of psychological mindedness, an important element of mental health [23].

Alongside attachment and mentalizing, two significant developmental processes associated with mental health, a person’s capacity for epistemic trust (the trust in socially conveyed knowledge or information) is also rooted in secure attachment. Epistemic trust (ET) refers to humans’ capacity to identify the knowledge conveyed by others as personally relevant and applicable to various contexts, thereby making it memorable and integrated into their knowledge schemas [24]. This capacity for mentalizing and recognizing the trustworthiness of the information source is considered crucial for how a child establishes the relationship between their internal and external world [25]. This could potentially account for some developmental vulnerabilities to psychopathology, particularly more severe mental disorders such as BPD [26, 27].

A questionnaire measure proposed by Campbell et al. [28] captures this epistemic social system using three factors: epistemic trust, epistemic mistrust, and epistemic credulity. Epistemic trust mirrors an adaptive predisposition in positive social circumstances where the individual is open to social learning opportunities within relationships, promoting psychological resilience. Conversely, epistemic mistrust implies a tendency to treat all information sources as unreliable or ill-intentioned, possibly leading to a rejection of communication influences from others. Epistemic credulity represents a notable lack of vigilance and discernment, indicating a general lack of clarity about one’s position and making individuals susceptible to misinformation and exploitation [28]. For convenience, in the text below we will refer to these dimensions as trust, mistrust and credulity.

In a study examining the associations of the three-dimensional ET scale with childhood trauma, attachment, mentalizing, and adult mental health symptoms, Campbell et al. [28] reported that mistrust and credulity were positively related to insecure attachment styles and mentalizing difficulties. Meanwhile, trust was negatively, albeit less robustly, associated with insecure attachment. Interestingly, higher indices of psychopathology were associated with mistrust and credulity, while trust was not associated with reduced mental health symptoms. Based on these findings, the authors underscored the role of an epistemic stance characterized by a propensity to mistrust and misjudge trustworthiness (credulity) as increasing the developmental risk of mental health problems. Their conclusion suggests that rather than trust, it might be lower levels of mistrust and credulity that are key factors in promoting resilience.

While current findings emphasize the potentially critical role of mentalizing or RF in both typical and atypical development, certain aspects of this construct remain largely unexplored. Existing research on mentalizing’s role in non-clinical populations is scarce and offers inconclusive results, with these limited findings further constrained by measurement concerns [29].

Much of the data has been gathered using two interview-based measures: the Reflective Functioning Scale (RFS) [30], applied to the Adult Attachment Interview (AAI) [31], and the Parent Development Interview [32], applied to an interview about parenting and experiences of interactions with a child. Nevertheless, the practical application of the RFS is limited due to issues of time intensity, labor-intensity, and the requirement for highly trained raters, which restricts its usage to smaller sample sizes only [33].

Additionally, other studies have utilized validated self-report measures developed to assess constructs related to mentalizing, such as mindfulness, perspective-taking, empathy, theory of mind, alexithymia, and psychological mindedness [9]. Although these measures offer statistical reliability and are easier to use, they only indirectly assess mentalizing and originate from different theoretical backgrounds, which further limits their applicability [29]. Thus, while advancements have been made in understanding the role and implications of mentalizing in human development and psychopathology, further exploration and refinement of its measurement tools remain a pressing requirement.

To tackle the aforementioned issues surrounding the measurement of mentalizing, a number of psychometrically sound instruments assessing RF has been developed: the Parental Reflective Functioning Questionnaire (PRFQ) [34], which assesses PRF, the Mentalization Questionnaire (MZQ) [35], the Mentalization Scale (MentS) [36], the Mental States Certainty Questionnaire (CAMSQ) [37], the Multidimensional Mentalization Questionnaire (MMQ) [38], and the Reflective Functioning Questionnaire (RFQ) which appears in a number of versions, developed from a 54-item questionnaire (RFQ-54) [39] (the RFQ-8 [9], the RFQ-6 [40, 41], the RFQ-15 [42] and the RFQ-18 [12]), all assessing RF in adults. Some modified versions of the RFQ have been developed to assess RF in adolescents: the 8-item Reflective Functioning Questionnaire for Youth (RFQY) [29], the 23-item RFQY Scale B [43], and the 6-item RFQY Scale B [43].

The RFQ underwent testing for reliability and validity in both clinical and nonclinical adult samples in the UK [9, 39]. In their study, Fonagy et al. [9] identified two subscales in the RFQ-8: Certainty about mental states (RFQ_C) and uncertainty about mental states (RFQ_U). Extreme scores on these two subscales represent two different forms of mentalizing impairments, termed hypermentalizing and hypomentalizing, respectively, which are considered common in several mental disorders, including but not limited to BPD, depression, and anxiety [8, 9, 11]. Hypermentalizing refers to a tendency to develop inaccurate models of one’s own mind and that of others, often reflected in long, excessively detailed accounts that bear little to no relationship to observable reality. In contrast, hypomentalizing refers to a predilection for concrete thinking, indicating an inability to conceive complex models of one’s own mind or that of others.

The RFQ-8 has been widely used in different populations and has been translated in many languages. Its psychometric properties have been evaluated in general population in English, French, Italian, Greek, German, Polish and Spanish; in clinical samples, e.g., in patients with personality disorders, and other psychiatric patients, in English, Italian, German and Spanish; and in type 1 diabetes adults in Greek [9, 29, 40, 41, 44–47]. The two-factor structure of the original RFQ-8 was further confirmed by some of these studies, thereby substantiating the instrument’s reliability and validity [29, 44, 45]. It should be noted that while longer versions of the RFQ (RFQ-54) have been used, maintaining the same two-factor structure (RFQ_C, RFQ_U), these versions lack extensive studies confirming their reliability and validity (e.g., [48]).

Interestingly, a recent study by Müller et al. [41] critically evaluated the RFQ and questioned the validity of the widely accepted eight-item measure, arguing that the RFQ is likely to assess a single dimension related to hypomentalizing but may fall short in capturing maladaptive forms of hypermentalizing; the concerns about the factor structure of the RFQ and the validity of the RFQ_C scale were connected to the double-scoring procedure which seemed to cause problems in factor analysis. Therefore, a change in scoring procedure was proposed, maintaining the scoring previously used in the RFQ design process (i.e., 1, 2, 3, 4, 5, 6, 7), except for item 7, reversely scored [40, 41]. Moreover, further evidence based on exploratory factor analysis (EFA) and confirmatory factor analysis (CFA) suggested that a one-factor model can sufficiently explain the observed covariation of the responses to RFQ-8 items when using this new way of scoring [40, 41, 46, 47]. Aditionally, given that neither RFQ-8 certainty pole (using new proposed way of scoring), nor its certainty scale (using the previously proposed way of scoring) showed positive associations with indications of psychopathology when testing for U shape [40, 41] or linear correlations [9, 15, 46, 49–53], it has been suggested that the RFQ-8 can not sufficiently measure hypermentalization [41, 47, 48]. Within this context, the unidimensionality of the RFQ-8 has been proposed as a better solution [40, 41, 46, 47]. In the same line of thinking, Desatnik and colleagues [54], in a study involving a sample of adolescents and young adults, used a 15-item unidimensional coding version of the RFQ, measuring strength or weakness in mentalizing, as more suitable for assessing mentalizing in non-clinical samples.

Nevertheless, in a study involving an adolescent sample, where a longer, 46-item version of the RFQ for youth was used [55], three factors were identified: uncertainty/confusion about mental states, interest/curiosity about mental states, and excessive certainty about mental states. These findings indicate that while the RFQ represents a promising tool for the assessment of mentalizing, further studies are needed to refine its structure and validate its application across various contexts.

The present study seeks to validate the full 54-item version of the RFQ, with the anticipation of corroborating a two-factor structure that mirrors subjective uncertainty or certainty about the relationships between mental states and behaviors. This hypothesis is grounded in previous research [9, 29, 44]. Additionally, we aim to investigate the correlations between the RFQ and measures of attachment styles (AttS), epistemic trust (ET), emotion regulation (ER) strategies, and psychological mindedness (PM)–variables that have theoretical and empirical connections to RF [3, 4, 20, 21, 28]

We anticipate positive relationships between the degree of certainty about mental states (RFQ_C) and trust, ER (specifically, reappraisal), and PM. Conversely, we predict negative associations between the degree of certainty about mental states (RFQ_C) and insecure AttS, epistemic mistrust and credulity, ER (specifically, suppression), and PM. Moreover, we propose a reverse pattern of correlations between these variables and the degree of uncertainty about mental states (RFQ_U). In alignment with the tradition of attachment research [3–5], we plan to employ a person-centered approach–cluster analysis–to examine the contribution of RF, along with ET, ER, and PM, to attachment profile classifications. We foresee three profiles corresponding to secure, avoidant, and anxious attachment styles. This comprehensive examination should provide a more nuanced understanding of how these variables interact and their potential implications for mental health and psychopathology.

In accordance with the existing literature [7, 9, 48], we employed anxiety, depression, and BPD clinical measures as indicators of criterion validity. We hypothesized that scores reflecting both uncertainty (RFQ_U) and certainty (RFQ_C) about mental states would be associated, in a positive and negative pattern respectively, with probable clinical levels of anxiety, depression, and BPD symptoms. These relationships are expected given prior research demonstrating connections between impaired mentalizing and various mental health conditions, including anxiety, depression, and BPD. This part of our study will further provide valuable insights into the potential clinical relevance and utility of the RFQ in identifying or predicting these prevalent mental health concerns.

## Methods

### Participants and procedures

The sample consisted of 875 participants recruited between October 3, 2020 and April 30, 2021 through social media or university advertisements from an urban community of Greek adults (89% female, 11% male) ranging in age from 17 to 72. The mean and median ages were 28.5 and 22, respectively. Inclusion criteria were being over 17 years of age and being a native Greek speaker. The majority of the sample (72%) were university students.

Participants completed a battery of online questionnaires, accessed through their university email address. Additionally, 32% of the participants were recruited via social media and were presented with the same set of questionnaires. Participation was voluntary, and all participants provided written informed consent prior to their involvement.

This study received ethical approval from the University of Ioannina Research Ethics Committee (35299/30-09-2020). The study was conducted in accordance with ethical standards and guidelines, ensuring the protection of participants’ rights, privacy, and well-being. All data collected was kept confidential and used solely for the purposes of this research.

### Measures

The RFQ [9, 39] was employed to gauge reflective functioning, an operationalized form of mentalizing. The items on the questionnaire were translated into Greek and then back-translated to English to ensure the preservation of their meaning. The RFQ is a 54-item tool that measures mentalizing abilities, evaluated through a participant’s responses to self-description questions about mental processes related to themselves and others.

The RFQ was designed to capture two dimensions of mentalizing with two subscales: Certainty about mental states (RFQ_C) and uncertainty about mental states (RFQ_U). In developing the RFQ, two types of items were designed in relation to their scoring system: some used a median-scoring method (extreme answers reflected lower scores, while less extreme answers- i.e., reflecting genuine mentalization- received the highest scores), and the others, the so called Scale B items [56], used a polar-scoring method (stronger agreement- or disagreement in case of inverted items- yielded higher RF scores). Only median-scored items were considered for further development of the questionnaire, but, as extreme answers for these items conflated hypermentalization and hypomentalization, it was decided to convert them into polar-scored items, using a double-scoring system (scoring them in two opposite directions to reflect hypomentalization and hypermentalization, respectively).

In this paper, in line with the study by Duval et al. [55], where the RFQ-Y was used, each item of the RFQ-54 was scored on a 7-point Likert-type scale, ranging from "strongly disagree" to "strongly agree", and no recoding was applied. All 54 items of the questionnaire (including RFQ_U and RFQ_C) were thus scored using the same Likert scale.

The Epistemic Trust, Mistrust and Credulity Questionnaire (ETMCQ) [28] is a 18-item measure which has not been previously used in Greek population. However, previous studies have indicated appropriate psychometric properties [28]. The questionnaire consists of three subscales: Epistemic trust, epistemic credulity, and epistemic mistrust. Each subscale includes 6 items, with responses rated on a 7-point Likert scale, from “strongly disagree” (= 1) to “strongly agree” (= 7). Positive correlations of mistrust and credulty with mentalizing difficulties (i.e., RFQ_U) have been reported [28]. In this study, Cronbach’s α for each subscale was = .76, .75, .57, respectively.

The Emotion Regulation Questionnaire (ERQ) [57] was employed to measure cognitive emotion regulation. It is a 10-item measure, used in Greek general population [58], that assesses two predominant emotion regulation strategies: reappraisal (6 items) and expressive suppression (4 items). Responses are given on a 7-point Likert scale ranging from “strongly disagree” (= 1) to “strongly agree” (= 7). Supression has been reported to be positively related to maternal pre-mentalizing (i.e., a non-mentalizing mode), a dimension of Parental Reflective Functioning Questionnaire (PRFQ), whereas reappraisal has been negatively correlated with the specific dimension of the PRFQ [22]. The scales have demonstrated reliability and good convergent validity [57–59]. In this study, Cronbach’s α was = .85 for reappraisal, and = .76 for suppression.

The Experiences in Close Relationships-Revised (ECR-RD12) [60] is a 12-item measure, used in Greek general population [61]. It assesses individual differences with respect to (a) attachment-related anxiety and (b) attachment-related avoidance (6 items for each subscale). Items are rated on a 7-point Likert scale, ranging from “strongly disagree” (= 1) to “strongly agree” (= 7). Positive correlations between attachment- related anxiety, avoidance and RFQ_U and negative correlations between the two dimensions of insecure attachment and RFQ_C (8-item version) have been reported [16]. This measure has demonstrated adequate psychometric properties and is often used to explore individual differences in attachment experiences [60–62]. In this study, Cronbach’s α was = .83 for anxiety, and = .80 for avoidance.

The Psychological Mindedness Scale (PMS) [63] was used to assess participants’ overall capacity for psychological mindedness, which may be expected to correlate with reflective functioning. It has not been previously used in Greek population. However, previous studies have indicated appropriate psychometric properties [63–65]. This 45-item scale responses are rated on a 4-point scale, ranging from “strongly disagree” (= 1) to “strongly agree” (= 4). A significant positive correlation has been reported between the PMS and the RFQ (a 15-item unidimensional coding version) [54]. In this study, the scale’s internal consistency was adequate (Cronbach’s α = .84).

The Beck Anxiety Inventory (BAI) [66] is a 21-item measure used to gauge the discomfort level participants experienced over the last month due to common anxiety symptoms. Scores are given on a 4-point scale, ranging from “not at all” (= 0) to “severely” (= 3). This instrument has shown strong psychometric qualities and a well-validated clinical cutoff [66–68]; particular cut-off scores have been suggested in the use of the measure (scores lower than 7 point to minimal anxiety; 7 to 15 points indicate mild anxiety; 15 to 25 points are compatible with moderate anxiety, while scores between 26 and 63 point to severe anxiety) [69].

The Beck Depression Inventory (BDI) [70] is a 21-item measure assessing depression symptoms and attitudes over the past week. Scores are provided on a 4-point scale, ranging from 0 to 3. This tool has demonstrated robust psychometric properties and a well-validated clinical cutoff [71, 72]; particular cut-off scores have been suggested in the use of the instrument (scores lower than 10 point to absence of depression; 10 to 20 points indicate mild depression, while scores higher than 20 point to depression) [73].

The Borderline Personality Inventory (BPI) [74], a 54-item true/false questionnaire, was used to measure participants’ overall level of borderline features. It has been used in community studies to identify probable diagnostic cases [9, 74, 75]; a particular cut-off score has been suggested in the use of the instrument (scores of 10 and above can be recommended as a cutoff point) [74].

### Data analysis

As a part of the validation process, the English version of the RFQ (54-item version) was initially translated into Greek by two native Greek speakers, following the guidelines of the International Test Commission (ITC) for test adaptation [76]. Two native English speakers then back-translated the Greek version of the RFQ into English. Any minor discrepancies noticed during the back-translation were duly corrected.

Subsequently, the analysis focused on studying the psychometric properties of the RFQ, evaluating its reliability and validity in the Greek-speaking population. For this purpose, we employed various statistical procedures, conducting, for example, exploratory (EFA) and confirmatory factor analysis (CFA), and calculating indices such as the average variance extracted and Cronbach’s alpha, among others.

In the beginning, to assess the latent structure of the RFQ, we performed a series of EFAs on training samples (using ordinary least squares for estimating model parameters, and promax rotation), which helped us in deciding: (1) the number of latent factors, and (2) which items were problematic and needed to be excluded from our analysis. The decision about the number of factors was guided by a combination of criteria provided by the parallel analysis, scree test, Velicer MAP, and Very Simple Structure (e.g., [77–80]). Furthermore, the primary reasons for excluding an item were its problematic loadings (low and/or cross loadings, i.e., an item with maximum loading less than 0.35, or two loadings larger than 0.35), along with theoretical (whether its exclusion made conceptual gaps in the instrument) and other statistical arguments (whether it had a positive contribution to the reliability indices, such as Cronbach’s alpha, or the value of Kaiser’s single-variable measure of sampling adequacy, MSA).

Considering all items were on an ordinal scale, we employed methods suitable for quantitative and qualitative variables. Accordingly, for the correlation matrices used in the analysis (e.g., on EFA), we considered either the Spearman correlation coefficient, the polychoric correlation, or the Pearson correlation, all of which delivered quite similar results.

Following the EFAs on training samples, we validated our decisions by performing CFAs on testing samples (e.g., [81–85]). We assessed the fit of the CFA models (with parameter estimation executed by the weighted least squares method) on our data using the following indices: Comparative Fit (CFI), Normed Fit (NFI), Goodness-of-Fit (GFI), Adjusted Goodness-of-Fit (AGFI), Tucker-Lewis Index (TLI), Root Mean Square Error Approximation (RMSEA) (along with the p-value for testing whether RMSEA is less than or equal to 0.05 or not), and Standardized Root Mean Square Residual (SRMR). It is noted that AGFI, TLI, NFI, GFI, and CFI were considered to have acceptable values, if they were larger than 0.90, whereas RMSEA and SRMR needed to be quite small (ideally, less than 0.05).

The robustness of the suggested latent structure of the RFQ or the effect of the sample size on its latent structure was further evaluated by implementing the following procedure:

First, we selected a random sample of size n from our total sample. We varied n to be 100, 200, 300, 400, 500, 600, or 700. Then, we executed an EFA on the selected sample, verifying whether the proposed solution was upheld and identifying the largest and second-largest loadings (in absolute values). We considered the solution confirmed if the items’ largest loadings were larger than 0.35 and found on the suggested/correct factors, while no other loadings larger than 0.35 were met. This procedure was repeated 100 times for each different sample size n. Furthermore, we took into account the values of the fit indices of the CFA and Cronbach’s alpha.

We also tested the factorial invariance of the RFQ (configural, weak, and strong invariance) across three age groups and sex. The construct validity was assessed by observing the correlations between the RFQ and the ETMCQ, ECR, ERQ, and PMS (we also evaluated the psychometric properties of all measures).

Moreover, we used decision tree models to gain a deeper understanding of the RFQ’s psychometric properties in relation to the groups derived from a set of clinical measures. Cluster analysis was also used for classifying respondents into homogeneous groups based on their responses to the RFQ, ETMCQ, ECR, ERQ, and PMS. We employed the “NbClust” package [86] provided by the R-project [87] to determine the number of underlying clusters in our data. Specifically, we considered five distance measures (euclidean, maximum, manhattan, Canberra, and minkowski) and six clustering methods (kmeans, ward.D, ward.D2, single, complete, and average). We evaluated any solution with a number of clusters ranging from 2 to 10. Consequently, for each combination of clustering methods and distances (thirty in total), an optimal clustering solution was provided following the majority rule provided by this package. The best clustering solution among the suggested was determined based on statistical and theoretical arguments.

The entire data analysis was performed using the standardized factor scores, computed by the CFA and the empirical Bayes modal approach found in the “lavPredict” function provided by the R-project (e.g., [88]).

In addition to the procedures already mentioned, our data analysis process included a thorough examination of our dataset for outliers or response profiles that significantly deviated from the majority, such as those from careless responders. For example, we identified and removed responses or cases where there was a surprisingly large number of consecutive similar answers to the items. This data cleansing process aimed to ensure the integrity and quality of our dataset. Following this procedure, our final sample size was n = 833. The data analysis was executed using R-project [87] and SPSS 29.0 [89].

## Results

### A. Psychometric properties of the RFQ

Our analysis of the RFQ suggests a compelling three-factor latent structure, comprised of 31 items from the original set of 54. Using a combination of EFA on randomly selected training samples and CFA for validation on testing samples, in addition to theoretical and statistical arguments, we present our case for the latent structure of the RFQ (Table 1). The identified factors are characterized as “excessive certainty”, “interest/curiosity”, and “uncertainty/confusion”.

**Table 1:** The RFQ items and the deduced factor structure.

Factor*	Item	
	1	People’s thoughts are a mystery to me
**1**	**2**	**It’s easy for me to figure out what someone else is thinking or feeling**
	3	My picture of my parents changes as I change
	4	I worry a great deal about what people are thinking and feeling
**2**	**5**	**I pay attention to the impact of my actions on others’ feelings**
	6	It takes me a long time to understand other people’s thoughts and feelings
**1**	**7**	**I know exactly what my close friends are thinking**
	8	I always know what I feel
	9	How I feel can easily affect how I understand someone else’s behaviour
**1**	**10**	**I can tell how someone is feeling by looking at their eyes**
**3**	**11**	**I realise that I can sometimes misunderstand my best friends’ reactions**
**3**	**12**	**I often get confused about what I am feeling**
	13	I wonder what my dreams mean
**1**	**14**	**Understanding what’s on someone else’s mind is never difficult for me**
	15	I believe that my parents’ behaviour towards me should not be explained by how they were brought up
**3**	**16**	**I don’t always know why I do what I do**
	17	I have noticed that people often give advice to others that they actually wish to follow themselves
	18	It’s really hard for me to figure out what goes on in other people’s heads
	19	Other people tell me I’m a good listener
**3**	**20**	**When I get angry, I say things without really knowing why I am saying them**
	21	I’m often curious about the meaning behind others’ actions
	22	I really struggle to make sense of other people’s feelings
**3**	**23**	**I often have to force people to do what I want them to do**
**3**	**24**	**Those close to me often seem to find it difficult to understand why I do things**
**3**	**25**	**I feel that, if I am not careful, I could intrude into another person’s life**
**3**	**26**	**Other people’s thoughts and feelings are confusing to me**
**1**	**27**	**I can mostly predict what someone else will do**
**3**	**28**	**Strong feelings often cloud my thinking**
	29	In order to know exactly how someone is feeling, I have found that I need to ask them
**1**	**30**	**My intuition about a person is hardly ever wrong**
**2**	**31**	**I believe that people can see a situation very differently based on their own beliefs and experiences**
**3**	**32**	**Sometimes I find myself saying things and I have no idea why I said them**
**2**	**33**	**I like to think about the reasons behind my actions**
**1**	**34**	**I normally have a good idea of what is on other people’s minds**
	35	I trust my feelings
**3**	**36**	**When I get angry, I say things that I later regret**
**3**	**37**	**I get confused when people talk about their feelings**
**1**	**38**	**I am a good mind reader**
**3**	**39**	**I frequently feel that my mind is empty**
	40	If I feel insecure, I can behave in ways that put others’ backs up
**3**	**41**	**I find it difficult to see other people’s points of view**
**1**	**42**	**I usually know exactly what other people are thinking**
	43	I anticipate that my feelings might change even about something I feel strongly about
**3**	**44**	**Sometimes I do things without really knowing why**
**2**	**45**	**I pay attention to my feelings**
**2**	**46**	**In an argument, I keep the other person’s point of view in mind**
**1**	**47**	**My gut feeling about what someone else is thinking is usually very accurate**
**2**	**48**	**Understanding the reasons for people’s actions helps me to forgive them**
	49	I believe that there is no RIGHT way of seeing any situation
	50	I am better guided by reason than by my gut
	51	I can’t remember much about when I was a child
	52	I believe there’s no point trying to guess what’s on someone else’s mind
	53	For me actions speak louder than words
	54	I believe other people are too confusing to bother figuring out

Items presented in bold comprise the suggested three factor solution (note that 31 out of the initial 54 items were finally used).

*Factor 1: “Excessive certainty about mental states of others”, Factor 2: “Interest/Curiosity in mental states”, Factor 3: “Uncertainty/confusion about mental states”

CFA results substantiate the soundness of this three-factor model, displaying acceptable fit indices across the board: Comparative Fit Index (CFI) at 0.945, Normed Fit Index (NFI) at 0.921, Tucker-Lewis Index (TLI) at 0.940, Goodness of Fit Index (GFI) at 0.961, Adjusted Goodness of Fit Index (AGFI) at 0.955, Root Mean Square Error of Approximation (RMSEA) at 0.051 (p = 0.333), and Standardized Root Mean Square Residual (SRMR) at 0.061.

Support for internal consistency of each of the three factors was found via Cronbach’s alpha coefficients (0.876 for “excessive certainty”, 0.678 for “interest/curiosity”, and 0.870 for “uncertainty/confusion”). Convergent validity, as evidenced by the average variance extracted (AVE) for each factor (“excessive certainty” at 0.429, “interest/curiosity” at 0.272, and “uncertainty/confusion” at 0.339), met the Fornell-Larcker criterion [90].

To further bolster the robustness of our model, we conducted an extensive numerical investigation. Random samples of varying sizes (n = 100 to n = 700) were selected from the complete sample and an EFA was conducted. This process was repeated 100 times for each sample size. The results, illustrated in Table 2, reflect how often our model was confirmed in these iterations. For instance, at a sample size of n = 100, the first, second, and third factors were confirmed 72%, 60%, and 40% of the time, respectively. With sample sizes larger than n = 400, the factors were nearly always confirmed.

**Table 2:** The number of times the factors of our model was confirmed (Prop.), along with the mean values of the fit indices and Cronbach’s alpha, as they derived by the 100 randomly chosen samples of size n.

	Prop.			Alpha			Fit index						
	Factor			Factor			Mean						
N	1	2	3	1	2	3	CFI	NFI	TLI	GFI	AGFI	RMSEA (p)	SRMR
100	72	60	40	.873	.662	.871	.989	.830	.998	.909	.895	.014 (.941)	.098
200	92	93	69	.875	.676	.870	.968	.876	.966	.936	.927	.038 (.897)	.080
300	100	98	86	.877	.675	.870	.959	.897	.956	.947	.939	.044 (.813)	.072
400	100	99	92	.875	.675	.870	.955	.907	.951	.953	.946	.046 (.826)	.068
500	100	100	99	.876	.682	.870	.950	.911	.946	.955	.949	.048 (.668)	.066
600	100	100	100	.875	.676	.869	.948	.915	.944	.958	.951	.049 (.620)	.064
700	100	100	100	.876	.678	.870	.947	.919	.943	.960	.953	.050 (.532)	.062

**Fig 1:**
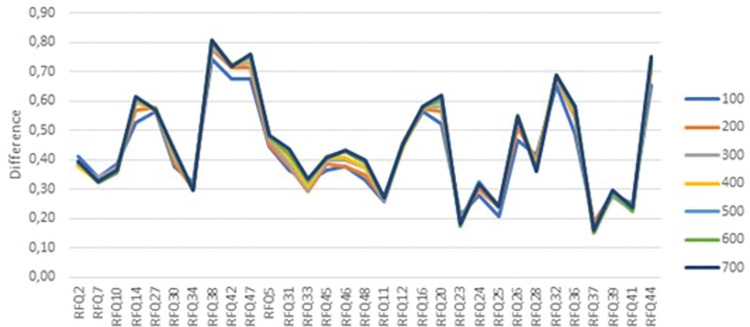
The mean difference between the largest and the second largest loadings (in absolute values) of each item and sample size (computed by 100 random samples).

**Fig 2:**
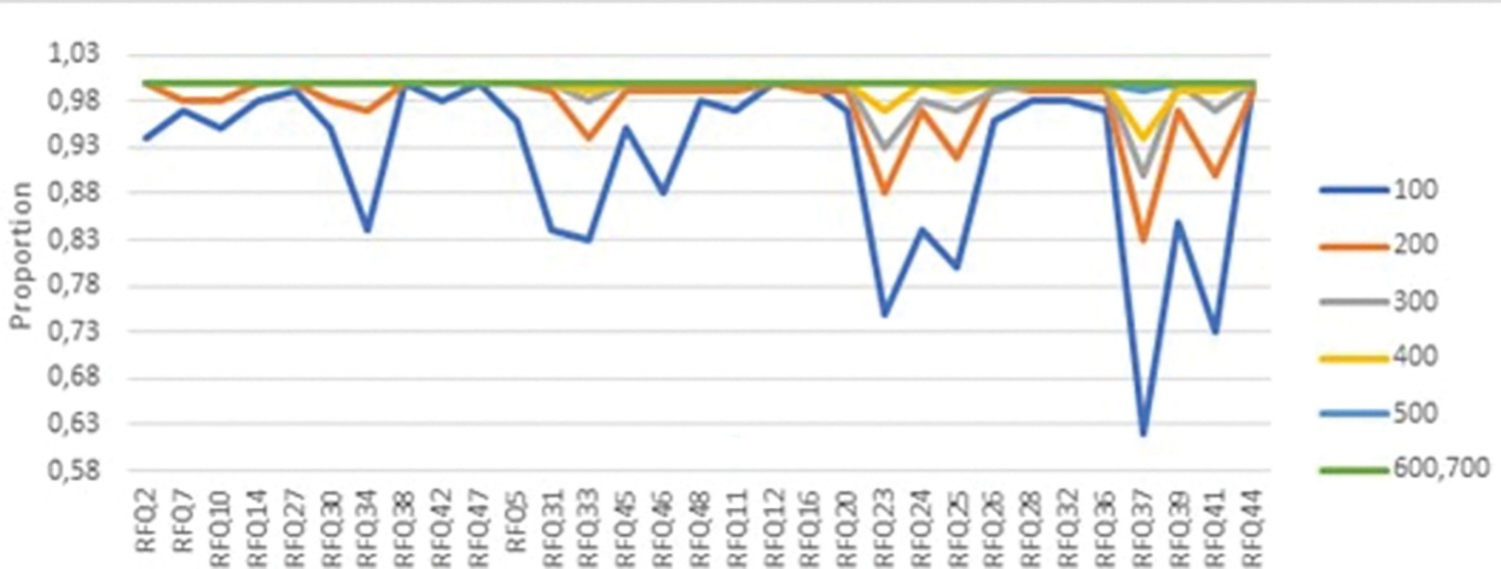
The proportion of times that an item loaded to the correct/intended factor, for each sample size (computed by 100 random samples).

Further, we examined the loadings of the items to assess potential cross-loadings. Fig 1 displays the mean differences between the largest and second-largest loadings (in absolute values), observed over the 100 iterations. Certain items, such as 23, 25, 37, and 41 from the third factor, showed relatively small mean differences and did not consistently load onto the third factor, particularly in the smaller sample sizes (Fig 2).

Our robustness check, based on calculating mean values of the CFA fit indices across the 100 iterations, revealed some limitations in confirming the model only with smaller sample sizes (especially n = 100 and n = 200), according to NFI and SRMR. However, despite some difficulties with smaller sample sizes, our findings largely corroborate the robustness and validity of the proposed three-factor model of the RFQ.

Moreover, we scrutinized the three-factor solution of the RFQ for measurement invariance across demographic variables, namely, sex and age groups. The age groups were demarcated into 18–19, 20–29, and 30 plus years old, reflecting developmental phases of interest based on recent findings from neuroscience research on mentalizing [2]. We examined three levels of measurement invariance: configural (the model structure is the same across groups), weak (also known as metric, the factor loadings are equal across groups), and strong (also known as scalar, the factor loadings and intercepts are equal across groups) (e.g., [83]). Our results indicate that weak invariance was supported for both sex (p = 0.331) and age groups (p = 0.313), indicating that the factor loadings were consistent across these groups. However, strong invariance was not upheld at the 0.05 level of significance, for both sex (p = 0.034) and age (p<0.001), suggesting that the intercepts varied across these groups.

To ensure the validity of our subsequent analyses, we verified the factorial structures of other scales used in our study, specifically, the ETMCQ, ECR, ERQ, and PMS. Table 3 presents the fit indices for these scales. While the latent structure of the first three scales was confirmed, some issues arose regarding RMSEA and SRMR fit indices for the ECR and PMS. In these analyses, several residual/error term covariances between items within the same subscales were freely estimated (three for the ETMCQ, two for the ECR, and thirteen for the PMS).

**Table 3:** Cronbach’s alpha and fit indices for the scales of the study.

Scale*	Alpha		CFI	NFI	TLI	GFI	AGFI	RMSEA (p)	SRMR
ETMCQ	Trust	0.764 0.751 0.577	.951	.931	.937	.979	.969	.052 (.325)	.056
	Credulity								
	Mistrust								
ECR	Anxious	0.838 0.804	.934	.925	.915	.970	.954	.086 (< .001)	.082
	Avoidant								
ERQ	Reappraisal	0.853 0.763	.987	.977	.983	.991	.986	.038 (.951)	.045
	Suppression								
PMS		0.848	.805	.763	.792	.895	.883	.065 (< .001)	.076

*Three, two and thirteen residual/error terms have been freely estimated, for Epistemic Trust, Attachment and Psychological Mindedness, respectively.

Invariance testing for these measures showed strong invariance for the ETMCQ across both sex and age groups. For the ECR, strong invariance was upheld across sex, while weak invariance was maintained across age groups. The ERQ demonstrated weak invariance across both sex and age groups. Notably, some difficulties were encountered in confirming the latent structure of the PMS due to subpar fit indices. Despite this, the measure’s internal consistency was adequate, with a Cronbach’s alpha of 0.848.

Finally, we computed Pearson’s correlations between the factor scores of the RFQ and the other scales, with the results outlined in Table 4. This process ensures a comprehensive understanding of the relationships and interactions between the RFQ’s factors and the constructs of the ETMCQ, ECR, ERQ, and PMS. More specifically, the uncertainty/confusion dimension showed significant, positive correlations with credulity, mistrust, and attachment- related anxiety; significant, though weak (i.e., a correlation less than .25, in absolute value), positive correlations with suppression and avoidance; and significant, although weak, negative correlations with psychological mindedness. On the other hand, certainty showed significant, but comparatively weak, negative correlations with credulity, attachment anxiety and avoidance; and significant but weak, positive correlations with reappraisal and PMS. Similarly, interest was correlated significantly and positively with trust, reappraisal and PMS while negatively and less strongly with suppression and avoidance.

**Table 4:** Pearson correlation coefficients between scales (computed on factor scores).

		RFQ			ETMCQ			ERQ		ECR	
		Certainty	Interest	Uncertainty	Trust	Credulity	Mistrust	Reappraisal	Suppression	Anxious	Avoidant
RFQ	Interest	**.560** ^**^	1	-0.041	**.368** ^**^	.030	.018	**.267** ^**^	-.180^**^	-.054	**-.275** ^**^
	Uncertainty	-.209^**^	-.041	1	.125^**^	**.527** ^**^	**.521** ^**^	-.089*	.180^**^	**.431** ^**^	.094^**^
ETMCQ	Trust	.081*	.368^**^	.125^**^	1	.225^**^	.001	**.281** ^**^	**-.331** ^**^	-.013	**-.341** ^**^
	Credulity	-.107^**^	.030	**.527** ^**^	.225^**^	1	**.878** ^**^	.007	.227^**^	**.487** ^**^	.032
	Mistrust	-.059	.018	.521^**^	.001	**.878** ^**^	1	-.090^**^	**.356** ^**^	**.557** ^**^	.136^**^
ERQ	Reappraisal	.179^**^	**.267** ^**^	-.089*	**.281** ^**^	.007	-.090^**^	1	-.073*	-.134^**^	-.175^**^
	Suppression	-.020	-.180^**^	.180^**^	**-.331** ^**^	.227^**^	**.356** ^**^	-.073*	1	**.300** ^**^	**.372** ^**^
ECR	Anxious	-.135^**^	-.054	**.431** ^**^	-.013	**.487** ^**^	**.557** ^**^	-.134^**^	**.300** ^**^	1	**.329** ^**^
	Avoidant	-.147^**^	**-.275** ^**^	.094^**^	**-.341** ^**^	.032	.136**	-.175^**^	**.372** ^**^	**.329** ^**^	1
PMS		.246^**^	**.517** ^**^	-.183^**^	**.594** ^**^	-.112^**^	**-.265** ^**^	**.320** ^**^	**-.454** ^**^	-.226^**^	**-.431** ^**^

*Correlation is significant at the 0.05 level

**Correlation is significant at the 0.01 level. Any correlation exceeding .25 (in absolute value) has been highlighted in bold.

### B. Cut-off grouping (clinical measures: BAI, BDI and BPI) and clustering

To evaluate potential clinical implications, participants were divided into groups according to their scores on the BAI, BDI, and BPI. These groupings were based on the pre-established cut-off scores from earlier research [69, 73, 74], resulting in four groups for anxiety, three for depression, and two for BPD.

Table 5 provides the descriptive statistics for the five scales across the groups created using these clinical measures. A multivariate version of the Kruskal-Wallis test (i.e., “kruskalmc” function in R-project; see also Ch. 8, [91]) was employed to conduct simultaneous pairwise comparisons across groups. However, it is crucial to interpret the mean differences with care, as none of the scales under investigation demonstrated strong measurement invariance across the groups defined by the clinical measures.

**Table 5:** Sample means and multiple pairwise comparisons; factorial invariance level is included in parentheses, across groups derived by BAI, BDI and BPI, respectively.

			RFQ (weak, weak, weak)			ETMCQ (strong, strong, weak)			ERQ (weak, weak, weak)		ECR (weak, weak, weak)		
		N	Certainty	Interest	Uncertainty	Trust	Credulity	Mistrust	Reappraisal	Suppression	Anxious	Avoidant	PMS
BAI	≤7	186	0.041	-0.024	-0.369	-0.020	-0.360	-0.196	0.016	-0.202	-0.470	-0.166	0.062
	(7,15]	213	-0.087	-0.037	-0.148	0.025	-0.245	-0.098	0.003	-0.113	-0.113	0.076	0.020
	(15,25]	209	0.059	0.032	-0.005	-0.026	0.032	0.017	0.060	0.059	-0.031	0.029	-0.024
	>25	225	-0.006	0.025	0.450	0.016	0.500	0.239	-0.072	0.219	0.524	0.038	-0.048
	Sig. pairs^1^		None	None	All (Except 2–3)	None	All (Except 1–2)	All (Except 1–2)	None	1–4,2–4	All (Except 2–3)	1–2	1–4
	Cor.^2^		-0.005	0.045	.377**	0.008	.352**	.399**	-0.046	.165**	.328**	.069*	-.127**
BDI	≤10	535	0.054	0.012	-0.196	-0.006	-0.238	-0.126	0.108	-0.150	-0.347	-0.109	0.042
	(10,20]	217	-0.030	0.021	0.183	0.062	0.318	0.171	-0.121	0.154	0.448	0.042	-0.016
	>20	81	-0.273	-0.138	0.801	-0.124	0.723	0.373	-0.391	0.581	1.091	0.608	-0.237
	Sig. pairs		1–3	2–3	All	None	All	All	1–2,1–3	All	All	1–3,2–3	1–3,2–3
	Cor.		-.106**	-0.012	.385**	0.026	.402**	.487**	-.145**	.224**	.502**	.196**	-.186**
BPI	≤10	707	0.032	0.020	-0.115	0.020	-0.119	-0.055	0.048	-0.107	-0.148	-0.060	0.030
	>10	126	-0.181	-0.113	0.647	-0.114	0.666	0.310	-0.271	0.602	0.832	0.335	-0.168
	Sig. pairs		All	All	All	None	All	All	All	All	All	All	All
	Cor.		-.131**	-0.020	.549**	0.013	.498**	.561**	-.117**	.294**	.499**	.212**	-.205**

^1^Sig. pairs: Statistically significant pairwise differences, at 0.05 level (based on multiple/simultaneously pairwise non-parametric comparisons).

^2^Spearman correlation coefficient.

*Correlation is significant at the 0.05 level

**Correlation is significant at the 0.01 level.

Due to this limitation, we further explored the relationships between the clinical measures and the five scales (in subscale level) using decision tree models with the exhaustive Chi-squared Automatic Interaction Detection (CHAID) method. This was done primarily for descriptive purposes, with the clinical groupings as the outcome variable and the factor scores of the scales as the independent variables (sex and age were also included).

**Fig 3:**
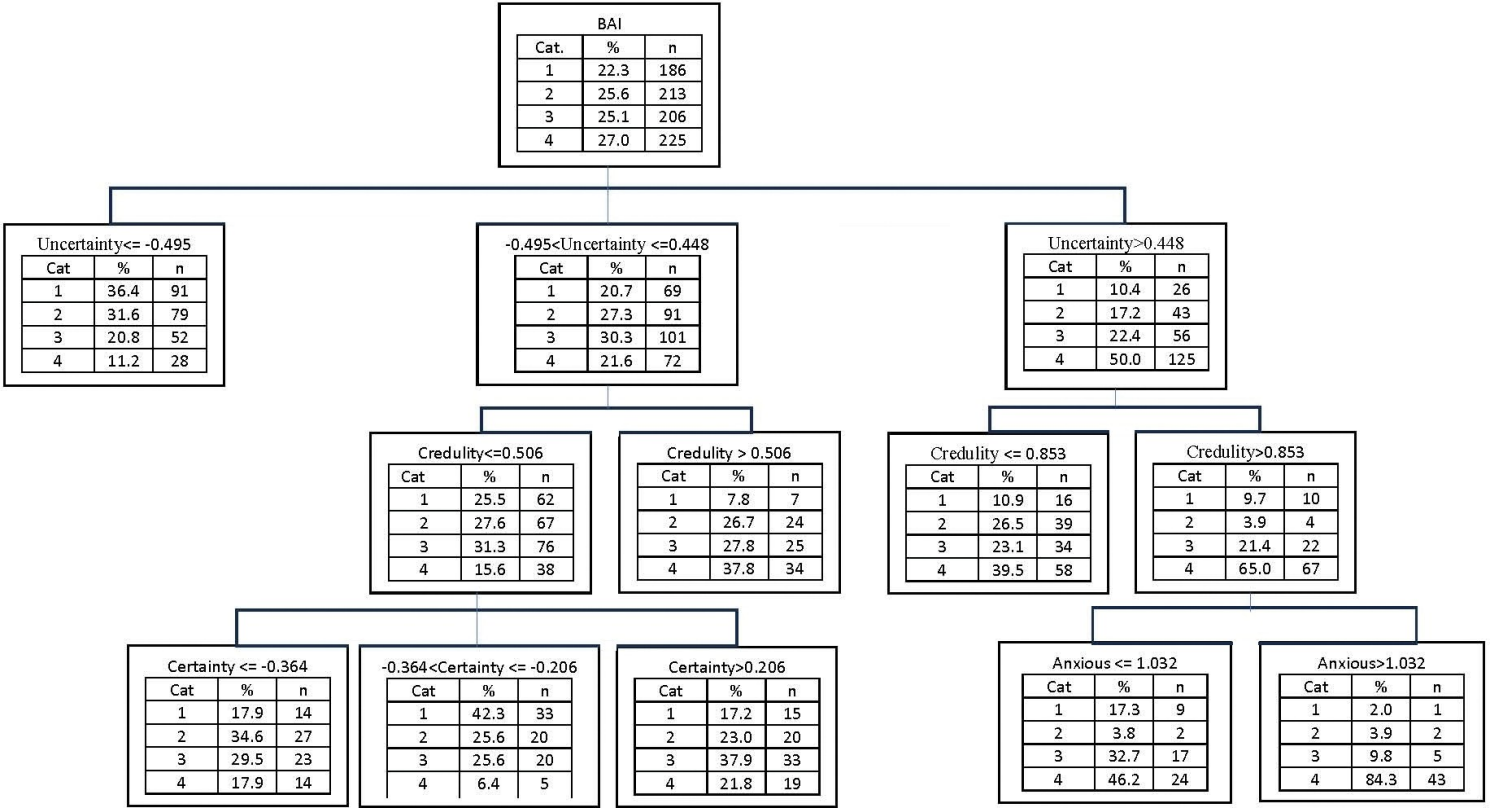
Decision tree model with BAI as dependent variable, and the subscales of RFQ, ETMCQ, ECR, ERQ, and PMS, as independent variables (using CHAID method).

Regarding BAI (Fig 3), although the overall proportion of participants in the highest anxiety group was 27%, a subset of participants demonstrated a proportion of 84.3% (43 out of 51 participants in this subset). These individuals were characterized by high scores in Uncertainty (>0.44), Credulity (>0.85), and Anxious attachment style (>1.03). Conversely, participants with medium Uncertainty scores (-0.49 to 0.44), low Credulity scores (<0.50), and medium Certainty scores (-0.36 to 0.21) showed a low proportion in the highest BAI group (6.4%).

**Fig 4:**
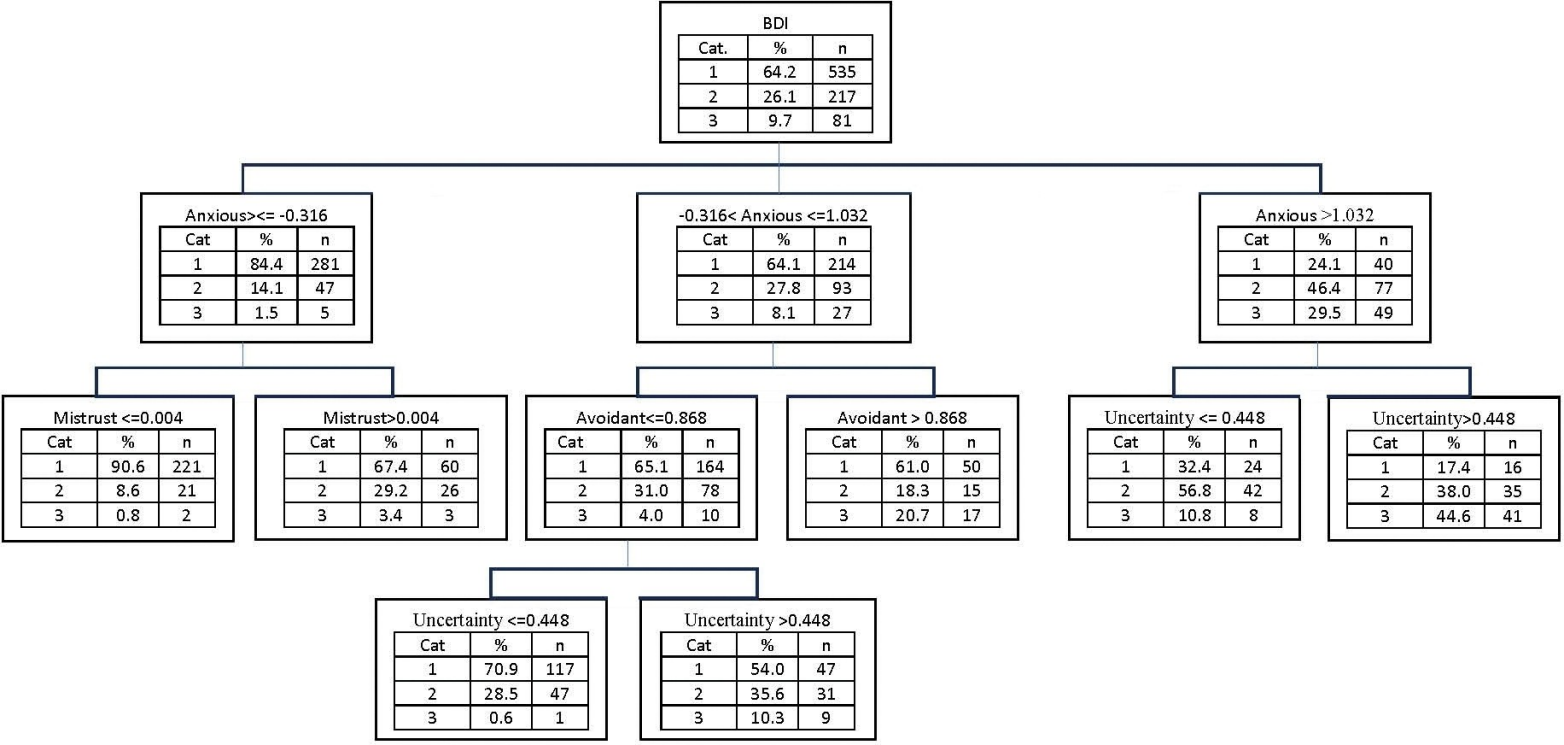
Decision tree model with BDI as dependent variable, and the subscales of RFQ, ETMCQ, ECR, ERQ, and PMS, as independent variables (using CHAID method).

Similarly, for the BDI grouping (Fig 4), the overall proportion of participants in the highest depression group was 9.7%. Yet, for participants with high scores in Anxious attachment style (>1.03) and Uncertainty (>0.44), this proportion rose to nearly 44.6% (41 out of 92 participants). On the other hand, this proportion was virtually zero (2 out of 244 participants) for those with low Anxious attachment style (<-0.31) and Mistrust (<0.004) scores. This was also the case for participants with medium Anxious attachment style scores (-0.31 to 1.03), low Avoidant attachment style scores (<0.87), and Uncertainty scores (<0.44).

**Fig 5:**
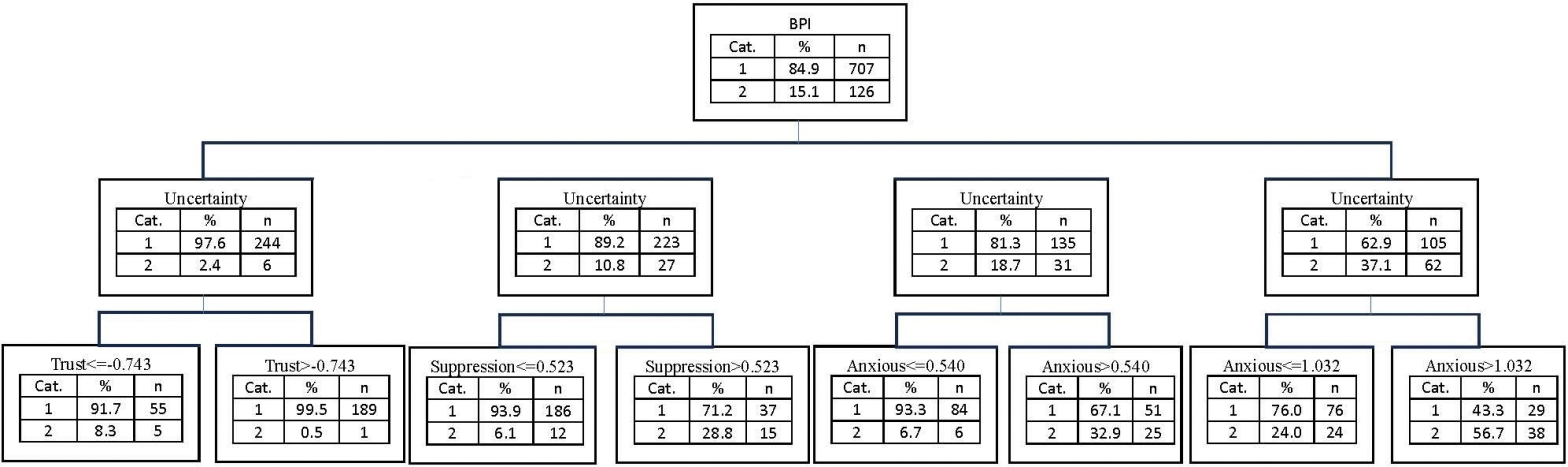
Decision tree model with BPI as dependent variable, and the subscales of RFQ, ETMCQ, ECR, ERQ, and PMS, as independent variables (using CHAID method).

As for the BPD categorization (Fig 5), the overall proportion of participants in the second group was 15.1%. This proportion could increase to 56.7% for those with high scores on Uncertainty and Anxious attachment style, or decrease to 0.5% for participants with low Uncertainty scores and high Trust scores.

Table 6 presents the findings from the cluster analysis. This process utilized the subscales (factor scores) of the RFQ, ETMCQ, ECR, ERQ, and PMS, to form distinct clusters within the sample. The mean values for each variable and group, as well as the statistical significance of the differences (using the “kruskalmc” function at a 0.05 significance level), are provided in the table.

**Table 6:** Cluster solution, sample means and multiple pairwise comparisons (if not mentioned otherwise, all the pairwise comparisons are significant at 0.05 level).

		Secure	Anxious	Avoidant
		1	2	3
	N	416	164	253
Used in cluster analysis	Excessive Certainty	0,33	-0,05	-0,51
	Interest/Curiosity*	0,38	0,30	-0,82
	Uncertainty Confusion	-0,48	0,83	0,26
	Trust	0,21	0,58	-0,72
	Credulity	-0,45	1,23	-0,06
	Mistrust	-0,53	1,13	0,14
	Reappraisal*	0,29	0,12	-0,56
	Suppression	-0,37	0,19	0,49
	Anxious	-0,48	0,75	0,31
	Avoidant	-0,39	-0,09	0,71
	Mindedness	0,55	0,06	-0,94
Outcomes	Anxiety	-0,23	0,52	0,05
	Depression	-0,34	0,44	0,28
	Borderline	-0,36	0,60	0,21
	Age**	29 years	24 years	28 years
	Sex (proportion of men) ***	11.5%	6.7%	19.4%

*The difference between groups 1 and 2 is not significant.

**The difference between groups 1 and 3 is not significant.

***All the differences are not significant.

The cluster analysis identified three distinct clusters within our data: “Secure”, “Anxious”, and “Avoidant”, comprised of 416, 164, and 253 respondents, respectively. Each group is characterized by their particular pattern of scores across the variables used in the analysis.

## Discussion

The primary aim of this study was to validate the factor structure of the Greek adaptation of the 54-item RFQ [9, 39] within a non-clinical adult population. Our findings propose a 31-item version of the RFQ (RFQ-31) with robust psychometric properties that are consistently effective across different age and gender categories.

Building upon previous research conducted on an adolescent sample [55], our study supports the adoption of a three-factor model. This structure delineates three validated subscales: excessive certainty, interest/curiosity, and uncertainty/confusion concerning mental states. The soundness of this instrument was upheld through both “conventional” and “heuristic” statistical methodologies.

Correlational analysis and cluster analysis were crucial components in the validation of the RFQ-31. Our study corroborates anticipated associations between the RFQ and measures of epistemic trust (ET), emotion regulation (ER), attachment styles (AttS), and psychological mindedness (PM). It further acknowledges the development of anticipated attachment classifications or profiles (derived based on ET, ER, AttS, and PM), thereby reinforcing the validity of the instrument.

The utilization of clinical measures of Anxiety, Depression, and BPD enabled us to investigate differences in RFQ-31 scores, as well as differences in ET, ER, AttS, and PM scores, across groups formed according to cut-off scores. Consistent with relevant literature [6–14], our results highlight the significant influence of excessive certainty, interest/curiosity, and uncertainty/confusion in distinguishing between BPD groups, and underscore the crucial role of uncertainty/confusion across all three clinical measures. Aligning with previous findings [28], our study underscores the inability of trust as a construct to differentiate groups across conditions of anxiety, depression, and BPD.

The present study lays the groundwork for validating the full-length RFQ by focusing on 31 out of the original 54 items. The findings underscore the validity and reliability of a significantly condensed version of the instrument, the RFQ-31, for usage across diverse age and gender groups.

While previous research has substantiated the configural invariance of certain RFQ versions [9, 29, 44] in clinical and non-clinical populations, our study presents weak invariance for the RFQ-31. This supports the argument that the RFQ-31, in comparison to previous versions like the RFQ-8, and the subscales of Certainty and Uncertainty [9], can serve as a more resilient instrument.

The three-factor model we propose is founded on 31 items and is associated with acceptable Cronbach’s alpha values for the three validated subscales. Specifically, the 31 items correspond to (a) excessive certainty about mental states (10 items), (b) interest/curiosity about mental states (6 items), and (c) uncertainty/confusion about mental states (15 items). Our proposed three-factor model contrasts with prior studies utilizing adult samples [9, 29, 44, 48], which have commonly reported a two-dimensional model of the mentalizing process, revealing two validated subscales: over-certainty (RFQ_C) and indiscriminate uncertainty (RFQ_U) about the mental states of oneself and others.

Interestingly, the inclusion of an "interest/curiosity" subscale in our model could suggest the presence of positive aspects of mentalizing, generative uncertainty, and an investigative approach towards mental states. This is particularly noteworthy in our non-clinical sample. This finding appears to refute a key criticism of previous studies, namely, the RFQ’s inability to "capture any positive qualities of mentalizing that may be distinct from the absence of non-mentalizing, such as curiosity about mental states, concern to integrate discrepant information about mental states, or effective use of support from others in conceiving or reconsidering mental states” ([92], p. 130). Therefore, our study suggests that a more comprehensive version of the instrument, like the RFQ-31, could potentially address this significant critique.

The validity of the RFQ-31 is substantiated further by its associations with measures of Epistemic Trust, Attachment styles, Emotion Regulation, and Psychological Mindedness, further strengthened by the validation of all the aforementioned instruments in our Greek sample. Consistent with the existing literature, the uncertainty/confusion dimension demonstrates significant and strong correlations with the variables examined in this study [4–6, 20, 21, 28, 92]. Perhaps less surprisingly, excessive certainty does not correlate with mistrust and suppression, potentially due to a lack of well-modulated doubt about the minds of oneself and others. The generation of productive uncertainty and healthy doubt requires the presence of trustworthy individuals capable of providing mirroring experiences. Moreover, the significant but less strong correlations found mainly between excessive certainty and the dimensions of the constructs examined suggest that such associations should be further investigated by future research. Nevertheless, the meaningful associations of our RFQ version’s dimensions with the other constructs examined, are further supported by our results discussed furtherdown.

Further evidence for the validity of the RFQ-31 comes from the exploration of differentiation between groups within three clinical measures: Anxiety [66], Depression [70] and BPD [74]. Our findings are in line with existing literature reporting poor mentalizing in those who score above the cut-off for BPD [8, 9, 11, 12, 55]. Individuals with high BPD scores reported lower scores on excessive certainty and interest/curiosity and higher scores on uncertainty/confusion.

In alignment with previous studies [7, 10, 18, 44, 93], our results underscore the crucial role of "uncertainty/confusion" in differentiating across groups within each of the three clinical measures: individuals high in anxiety, depression, and BPD tend to report high scores on uncertainty. Meanwhile, the dimensions of certainty and interest/curiosity fail to differentiate groups within the anxiety disorder category, though uncertainty as a mentalizing difficulty does distinguish between individuals with high and low scores on anxiety [14]. Overall, with regard to “excessive certainty” scale, our results on its negative associations with psychopathology dimensions, combined with its aforementioned positive correlations with the “adaptive” dimensions of the constructs examined, suggest the difficulties of the scale to assess a form of mentalization deficit, i.e. hypermentalizing or pretend- mode, challenging the capacity of our RFQ version to measure two different mentalization failures.

The robustness of the RFQ-31 was further evaluated through a comprehensive "heuristic" numerical analysis that combined Exploratory Factor Analysis, Confirmatory Factor Analysis, and reliability scores to assess the factor structure of the RFQ. This approach was necessitated due to inconsistent findings regarding the psychometric properties of different measures of the RFQ reported by previous studies [29, 41, 55]. Most of the items in our study loaded primarily on their "expected" factor, a finding that was bolstered by the large sample size. However, four items (items 23, 25, 37, and 41) should be interpreted with caution in smaller sample sizes. These items may reflect cultural differences between Greek and UK societies. Notably, Greek society retains features of a collectivist culture, which might manifest, to some degree, in the form of symbiotic interpersonal relationships. Greek citizens may feel less recognized as individuals and perceive less mentalization from their social system [94, 95]. These feelings may be exacerbated by the lingering effects of recent economic crises and the protracted "cost of living" crisis, which may foster caution towards others and inhibit perspective-taking.

Further validation of the RFQ-31 is substantiated by its alignment with the mentalizing-Attachment theoretical framework. Our cluster analysis lends a dual contribution to this framework: (a) the distribution of participants in each cluster corresponding to Secure, Avoidant, and Anxious attachment styles aligns with the proportions reported in existing literature, where 55%-65% are Secure, 22–30% are Avoidant, and 15%-20% are Anxious [96]; and (b) the variable scores for each profile, as reported by the individuals, align with the expected direction based on the attachment classification adopted in the analysis.

The age-related differences observed in this study corroborate recent findings from psychological research and neuroscience. The impacts of attachment on mental health and personal adjustment appear to decrease with age, with adults reporting more adaptive behavior patterns. This is supported by the maturation of the prefrontal cortex [97], which generally occurs later in life, around the age of 30, and could explain the lower mean age of anxiously attached participants in our sample.

Numerous studies have demonstrated that both mentalizing and attachment systems play a central role in emotion regulation and stress management and show high levels of functional connectivity at both the behavioral and neural levels [2]. The similar high scores on "reappraisal" and "interest/curiosity" as reported by Securely and Anxiously attached participants align with recent studies examining similar constructs [58].

Nonetheless, high scores on credulity, mistrust, and interest/curiosity in mental states among Anxiously attached individuals may signify a form of non-authentic, or “false”, adaptive behavior that lacks generative doubt, potentially compromising self-coherence. This hypothesis is bolstered by their high scores on anxiety, depression, and BPD measures, indicative of psychological instability and elements of psychopathology. As previous research suggests, "mentalizing impairments are transdiagnostic and transtheoretical vulnerability factors for psychopathology; temporary or chronic impairments in mentalizing are implicated in a wide range of psychological problems and disorders" ([2], p. 318).

Lastly, the apparent lack of gender differences in the three groups should be interpreted cautiously, given the overrepresentation of females in the university departments from which the majority of our subjects were recruited. Prior studies have identified gender differences in these areas [29].

To our knowledge, this study is the first to provide explicit empirical support for the theoretical associations between Attachment, Reflective Functioning, and Epistemic Trust. Our findings demonstrate that a significant portion of participants with high scores on anxiety, depression, and BPD also scored highly on excessive uncertainty, credulity, and anxious attachment [28, 98, 99]. These three variables appear to play a critical role in psychopathology, as measured by our clinical scales. Misperceptions of others’ behavior and one’s own mental states can lead anxiously attached individuals to desperate attempts for meaning. Severe impairments in mentalizing and non-mentalizing can stimulate an “epistemic hunger”, where the intense desire to feel recognized by others may amplify credulity when someone appears to offer the possibility of recognition ([92], p.187). Overall, this study supports previous findings revealing connections of uncertainty, mistrust and credulity with psychopathology. Such associations can be seen to corroborate theoretical underpinnings about social learning and psychopathology.

Our findings also suggest that suppression plays a significant role specifically in BPD. Among participants who scored moderately low on uncertainty and low on suppression, a considerable percentage also presented low scores on BPD. This is in alignment with previous studies that suggest core aspects of BPD are predicted by poor mentalizing associated with difficulties in emotion regulation [48]. Further, our study corroborates the existing literature linking impaired mentalizing and BPD [44, 55]; it appears that uncertainty is the primary factor contributing to differentiation between individuals who scored high and low on BPD.

## Conclusion

The current study presents a shortened version of the RFQ-54, comprised of 31 items. This condensed version proves to be a robust instrument, demonstrating superior psychometric properties in comparison to most other RFQ-based measures [9]. Our comprehensive investigation of the RFQ-31’s invariance, factor structure, and associations with a range of variables, alongside the use of psychopathology measures, strongly advocates for the RFQ-31 as a potent instrument in this field of study.

However, the study is not without limitations. The convenience sampling strategy resulted in an overrepresentation of women, university students, and young adults, which may limit the generalizability of the findings. Furthermore, this study used a community sample. A clinical sample for comparison, rather than using cut-off levels, would possibly give different results enabling us to draw different conclusions. For future research, it would be advisable to assess the psychometric properties of the RFQ-31 across different sample types, and particular caution should be exercised with the four items that displayed problematic effect sizes. Moreover, the difficulties of the “excessive certainty” scale of the instrument to assess hypermentalizing suggest that future studies should find ways to capture the full complexity of others forms of mentalizing such as pretend mode.

Nonetheless, the RFQ-31 presents a valuable tool for use within university student populations with higher confidence. Further research within this demographic could contribute meaningfully to the broad spectrum of research in higher education, deepening our understanding of mentalizing and its associated constructs.

## Supporting information

S1 File (CSV)
